# Epigenetic alterations in stem cell ageing—a promising target for age-reversing interventions?

**DOI:** 10.1093/bfgp/elab010

**Published:** 2021-03-19

**Authors:** Andromachi Pouikli, Peter Tessarz

**Keywords:** epigenetics, chromatin, ageing, stem cells

## Abstract

Ageing is accompanied by loss of tissue integrity and organismal homeostasis partly due to decline in stem cell function. The age-associated decrease in stem cell abundance and activity is often referred to as stem cell exhaustion and is considered one major hallmark of ageing. Importantly, stem cell proliferation and differentiation potential are tightly coupled to the cellular epigenetic state. Thus, research during the last years has started to investigate how the epigenome regulates stem cell function upon ageing. Here, we summarize the role of epigenetic regulation in stem cell fate decisions and we review the impact of age-related changes of the epigenome on stem cell activity. Finally, we discuss how targeted interventions on the epigenetic landscape might delay ageing and extend health-span.

## Introduction

Chromatin describes the macromolecular complex of proteins and DNA that can be found in the nucleus of every eukaryotic cell. It provides the scaffold for packaging the entire genetic material, facilitating its compaction and protecting DNA. The core of chromatin consists of the nucleosome. Research during the last decades has revealed that post-translational modifications on histones, which are the protein components of nucleosomes, and on DNA itself regulate gene expression and allow time- and tissue-controlled read-out of the genetic information [1, 2]. These studies have highlighted the contribution of chromatin architecture and dynamics during physiological development as well as upon tumorigenesis [3, 4]. Recent data also underscore the central role of epigenetics in the development and progression of ageing and age-related diseases, such as cardiovascular, neurodegenerative and metabolic disorders [5].

Adult stem cells play a vital role in tissue repair and regeneration; thus, they are essential for the maintenance of tissue homeostasis. Stem cell exhaustion, which describes the decline in the stem cell number and/or function, is one well-established hallmark of ageing. During the last years, the role of the epigenome in the regulation of adult stem cell activity, particularly in the context of ageing, has been the subject of increased scientific interest. Given the major contribution of stem cells in the maintenance of tissue integrity and homeostasis, investigating the mechanisms that govern stem cell ageing is of exceptional importance for interventions aiming at delaying or even preventing ageing and age-associated pathologies. However, stem cell ageing research is extremely challenging, mostly due to technical limitations, such as the isolation of pure stem cell populations in sufficient numbers. As a result, we still have only limited insight into the age-related changes of the stem cell epigenome. Nevertheless, the increasing progress in identifying suitable markers for purification of homogenous stem cell populations, the constant improvement of flow cytometers allowing cell sorting based on several markers simultaneously and, more importantly, the development of sensitive (epi-)genomic assays to study the chromatin landscape even at single-cell resolution has now made it possible to address these intriguing questions during the course of ageing. Here, we summarize the current knowledge on the role of epigenetics in the ageing process focusing on the impact of age-dependent alterations of the epigenome on stem cell physiology. We conclude our review discussing how careful interventions to the epigenetics-ageing axis might represent a potential way to alter stem cell fate decisions, favouring rejuvenation of aged stem cells and enhancing tissue homeostasis.

## Age-associated alterations of the epigenome

DNA methylation was discovered almost simultaneously with the identification of DNA as the genetic material [6, 7]. We know now that ~70% of cytosines are methylated genome-wide, as part of the dinucleotide motif CpG [8] and that DNA methylation at gene promoters correlates with transcriptional repression. The responsible mechanisms for the establishment and maintenance of DNA methylation as well as its role in transcriptional regulation are extensively discussed elsewhere [1, 9].

Given the early discovery of DNA methylation, it is not surprising that the first data on age-associated epigenetic modifications focused on the analysis of the DNA methylation profile in young and old individuals. Early studies showed that DNA methylation decreases with age in several organs in salmons, rats and mice [10–12]. Around ~25% of the CpG sites in mice exhibit age-related methylation changes across tissues, with the most prominent alterations observed in highly proliferative organs, such as the gut and the spleen [13]. Interestingly, the alterations of the DNA methylation profile occur progressively and linearly upon ageing, with similar rates between hyper- and hypomethylation; a phenomenon that has been described as epigenetic drift. The progressive modulation of the DNA methylation status with age explains the use of methylation patterns as a biomarker of ageing. Indeed, there are now available several global and tissue-specific epigenetic ageing clocks that are built on statistical models, taking into consideration the methylation level at specific genomic loci [14].

Apart from the changes in the DNA methylation profile, the chromatin landscape undergoes dramatic alterations upon ageing, including re-organization and loss of heterochromatic regions. In particular, reduction or redistribution of the transcriptional repressing H3K9me2/3 marks disrupts HP1 localization [12] and affects heterochromatin organization. Such heterochromatin rearrangements have been correlated with ageing in *C. elegans*, *D. melanogaster* and humans [15–17]. Similarly, epigenetic modifications in euchromatin have also been reported to change with age. Although this subject has been covered in detail elsewhere [18, 19], for the purpose of this review, we would like to discuss two important observations that highlight the fundamental role of an altered epigenetic landscape in the ageing process. Firstly, genetic manipulation of chromatin modifiers has been shown to elicit a profound effect on longevity in different model organisms; deletion of the chromatin-associated proteins ASH-2, WDR-5, and SET-2 leads to decreased H3K4me3 levels and subsequently, lifespan extension in *C. elegans* [20]. In contrast, mutations in *set1* and *met-1* genes, which encode for the enzymes that deposit H3K36me3, reduce lifespan of *S. cerevisiae* and *C. elegans*, respectively [21, 22]. Furthermore, in a landmark publication, Ocampo *et al*. [23] demonstrated that a transient and subtle induction of *Oct4*, *Sox2*, *Klf4* and *Myc* genes ameliorates progeria and improves regeneration of various murine tissues, via epigenetic remodelling. These examples indicate that epigenetic reprogramming has a beneficial effect on organismal function and is associated with extension of the health-span and lifespan.

Changes in epigenetic modifications represent one extensively studied hallmark of ageing. However, one important issue to consider when interpreting results from epigenetic studies in aged organisms is that so far most of them have been carried out in complex biological systems or tissues composed of multiple cell types. Thus, these data likely reflect the average of all the cell types found in each tissue. Therefore, the possibility that the epigenetic drift is also—at least in part—caused by a change in tissue composition with age cannot be excluded. To this end, development of methods to analyse DNA methylation and chromatin architecture at single-cell resolution and establishment of ChIP-like approaches which require low cell numbers, such as the CUT&RUN [24] and the CUT&Tag [25] technics, will considerably facilitate research on the role of epigenetics in ageing.

## Epigenetic regulation of fate decisions in adult stem cells

Adult stem cells have the dual capacity to proliferate and differentiate into specific lineages in response to various internal and external stimuli [26, 27]. Given that stem cells share the same genetic information with somatic cells, their epigenome and the associated transcriptional signature distinguish them from their differentiated counterparts. Importantly, the characteristic epigenetic profile of stem cells reflects their wide developmental potential. During the last years, the molecular mechanisms via which changes in the chromatin landscape control stem cell fate decisions have been the subject of intense scientific research, both in embryonic stem cells (ESCs) as well as in various adult stem cell populations.

**Figure 1:**
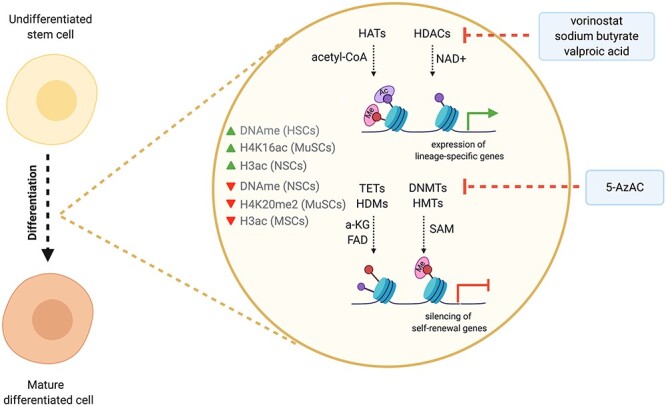
Epigenetic changes during stem cell differentiation. Overview of the changes that occur on the epigenome during differentiation of stem cells. DNA methylation increases in the differentiating HSCs, while it decreases upon NSCs differentiation. H3acetylation (H3ac) is higher in the differentiating NSCs, whereas it is reduced in MSCs during lineage commitment. Differentiating MuSCs exhibit high levels of H4K16ac and low levels of H4K20me2. Importantly, these changes occur at specific genomic loci, favouring the expression of lineage-specific genes and preventing expression of self-renewal and stemness-related genes. In blue boxes are indicated small molecules and metabolites that influence the activity of the respective epigenetic enzymes, affecting stem cell fate decisions.

As discussed above, DNA methylation at regulatory regions, such as gene promoters and enhancers, impacts stem cell fate decisions via altering the transcriptional output (Figure 1). For instance, differentiating ESCs display increased DNA methylation and high levels of H3K9me3 and H3K27me3, but low levels of H3K4me3 [28–30]. This increase in the transcriptional repressive marks promotes silencing of self-renewal genes and enhances lineage commitment. In contrast, regulatory regions of differentiation-associated genes are usually methylated in quiescent hair follicle stem cells (HFSCs), whereas they undergo a progressive, but profound de-methylation upon differentiation [31]. Likewise, neural stem cells (NSCs) display high DNA methylation, which is lost upon induction of differentiation, favouring efficient development of mature neurons [32]. The critical role of DNA and histone methylation in the regulation of stem cell function has also been investigated using various genetic models. For example, loss of the DNA methyltransferase 3A and/or 3B (DNMT3A/B) impairs the differentiation capacity of murine haematopoietic stem cells (HSCs) [33, 34]. Similarly, deletion of KMT5B, the enzyme that deposits H4K20me2, activates muscle stem cells (MuSCs) and forces them to differentiate. This leads to depletion of the MuSC population and impairs muscle integrity [36]. Furthermore, deletion of *Tet1*, the enzyme involved in DNA hydroxymethylation, regulates the expression of Wnt target genes in mouse intestinal stem cells (ISCs), altering their self-renewal capacity and influencing the integrity of the intestinal epithelium [35].

Histone acetylation is associated with activation of gene transcription and requires acetyl-CoA as the donor of the acetyl-group. Several studies have investigated the role of histone acetylation in different stem cell populations both in proliferating cells as well as during the course of differentiation. In mesenchymal stem cells (MSCs), elevated levels of global H3 acetylation (H3ac) correlate with enhanced stemness; indeed, in proliferating human MSCs increased H3ac promotes expression of core pluripotency genes, whereas osteogenic differentiation is accompanied by loss of global H3ac, leading to repression of self-renewal genes [37]. Consistently, histone deacetylation by SIRT3 is essential for efficient adipogenesis. In contrast, in mouse MuSCs elevated H4K16ac drives myogenic differentiation [38], whereas inhibition of histone deacetylases in NSCs is crucial for neurogenesis in mice and rats, favouring the development of both neuronal and glial lineages [39, 40]. In ISCs, nutritional interventions in the form of fasting or caloric restriction leads to elevated b-hydroxybutyrate levels and inhibition of histone deacetylases [41, 42]. These alterations influence ISC function via changing the H3K27ac abundance on promoters of genes involved in the Notch pathway [41]. Therefore, it is evident that the effect of histone acetylation on the regulation of the proliferation/differentiation balance depends heavily on the cell type and the modified residue.

Findings from epigenetic studies in adult stem cell populations have also led to the discovery of drugs, usually small molecules and metabolites, which can redirect—and even reprogram—stem cells towards specific lineages. For instance, vorinostat and sodium butyrate, two inhibitors of histone deacetylases that are commonly used in cancer treatment, arrest murine NSCs in the G1 phase and control cell cycle progression and differentiation [36]. Furthermore, Kohyama *et al*. [43] showed that treatment of mature osteoblasts with 5-azacytidine (5-AzaC), an inhibitor of DNA methylation, converts them to NSCs, via alterations in their epigenome and their gene expression profile. Similarly, osteocytes treated with valproic acid, a histone deacetylase inhibitor, are able to re-differentiate to functional NSCs [44]. In this context, it is worth mentioning that metabolism might represent a potential means to alter the epigenome and thus the stem cell identity. More specifically, a growing body of evidence suggests that chromatin interacts with metabolism in a direct and dynamic manner. Similar to the central role of acetyl-CoA in the establishment of histone acetylation marks, the metabolism-chromatin interaction is primarily mediated via intermediate metabolites, including α-ketoglutarate (α-KG) and S-adenosylmethionine (SAM). These metabolites serve as cofactors and substrates for epigenetic writers and erasers, modulating their activity and thus affecting stem cell fate decisions [45–48].

These are just a few examples—and by no means an exhaustive overview—that illustrate the tight control of stem cell function by epigenetics.

### Epigenetic changes in ageing stem cells

Age-dependent decline in stem cell number and function is observed virtually in all tissues and organs and is an important hallmark of ageing [49]. As discussed above, stem cells play a fundamental role in maintaining tissue homeostasis throughout the lifespan of an organism [50] and their potency is tightly linked with their epigenome. Therefore, it is not surprising that chromatin-mediated changes in stem cell function upon ageing have a profound impact not only on their resident tissue but ultimately on the physiology of the whole organism. The contribution of epigenetic modifications on stem cell ageing is underscored by genetic models. For instance, *Sirt7* knock out in mice is shown to drive the exit of HSCs from quiescence and to promote aberrant HSCs proliferation. This phenotype is reminiscent—at least in part—to that of aged HSCs. Mechanistically, this is achieved by loss of H3K56 deacetylation at the promoter of Wnt target genes [51]. Furthermore, in HFSCs, SIRT7 controls hair growth by regulating the transition from telogen to anagen during the hair-follicle life cycle. *Sirt7* is downregulated in aged HFSCs, which correlates with the well-known age-related loss and thinning of hair. Importantly, overexpression of *Sirt7* in HFSCs of older animals restores hair-growth. Mechanistically, this is mediated via deacetylation of the transcription factor NFATc1, which leads to its destabilization and the subsequent initiation of the hair follicle cycle [52]. For an extensive review of similar examples that highlight the impact of epigenetics on stem cell function during ageing, we refer the interested readers to recent reviews on this topic [53–55] and to Table 1.

**Table 1:** Summary of age-related epigenetic alterations in several stem cell types and their functional consequences on stem cell activity

	Stem cell type	Modification		Functional consequence	Ref.
Enzyme	HSC	DNMT1	▼	Lineage bias & self-renewal defects	[56–58]
		DNMT3A/B	▼	Arrest of HSC differentiation	[33]
		TET1	▼	Enhanced HSC self-renewal & myeloid lineage skewing	[59]
	MSCs	HDAC	▼	Senescence	[60]
		SIRT6	▼	Redox imbalance through H3K56ac on *Nrf2* promoter	[61]
	MuSCs	DNMT1	▼	Lineage bias & self-renewal defects	[62]
	NSCs	DNMT3A/B	▼	Arrest of NSC differentiation	[63]
		SIRT1	▼	Abnormal expansion of oligodendrocyte progenitors	[64]
	ISCs	DNMT1	▼	Lineage bias & self-renewal defects	[36]
		TET1	▼	Enhanced ISC proliferation	
	HFSCs	SIRT7	▼	Arrest of the hair follicle life-cycle transition from telogen to anagen	[52]
Histone modification	HSCs	H3K4me3 local broadening of peaks	▼	Broadening at genes involved in self-renewal & loss of differentiation	[65]
		H3K27ac	▼	Altered expression of tumour-suppressor genes	[66]
		H4K16ac diffuse pattern	▼	Myeloid lineage skewing & misformed nuclei	[67, 68]
	MSCs	H3ac & H4ac	▼	Impaired osteogenesis	[69]
		H3K9me3	▼		[70]
	MuSCs	H3K4me3	▼	Impaired stem cell function & chromatin remodelling	[71]
		H3K27me3	▲		
	NSCs	H3K27me3	▲	Inhibition of senescence-associated genes	[64]

But why does the chromatin landscape and the DNA and histone modification profile change upon ageing? Several studies in a wide range of organisms and cell types suggest that age-dependent DNA damage elicits permanent changes on the epigenome, redistributing chromatin-associated proteins and resetting the chromatin landscape. These alterations are not restored after repair of the DNA damage and have a profound impact on both stem cell and organismal function. For instance, in HFSCs, SIRT7, apart from regulating cell cycle, plays a significant role in the repair of DNA double-strand breaks (DSBs), via H3K18 deacetylation at the break site. This triggers the recruitment of the damage response factor 53BP1 to the DSB site. Thus, *Sirt7* deletion has dramatic consequences on the organismal function, resulting in shorter lifespan in mice [72]. In addition to DNA damage, other mechanisms contribute to the age-dependent changes in the epigenome, e.g. metabolic alterations that impact chromatin modifications [73], as metabolism and epigenome are tightly linked via the abundance of central metabolites [53]. Recent data also point towards an impact of age-related changes in cellular polarity on the epigenome [68].

While data from genetic approaches, like the examples described above, are essential to provide functional insights into the role of epigenetics during stem cell ageing, it is also vital to start incorporating age-associated changes in the DNA methylation profile, histone modification patterns and chromatin architecture in order to understand the general trends and identify commonly affected genes. Of note, these integrated studies should be performed in homogenous cell populations, ideally over the course of the lifetime and in both sexes to fully characterize the age-related changes as well as the complex underlying mechanisms. Although technically challenging, such studies will definitely shed light on the role of epigenetics in stem cell ageing.

**Figure 2:**
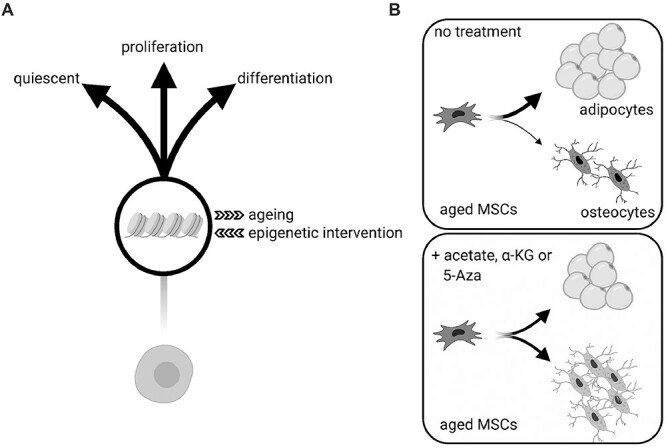
The epigenome and cell fate decisions. (A) In stem cells, the epigenome plays an important role in the regulation of fate decisions, which are heavily affected by age-associated epigenetic alterations. Targeting the ageing epigenome represents a potential tool to reverse these changes and re-establish the full potency of the stem cell population. (B) MSCs are used as an example to illustrate the effect of epigenetic interventions in stem cell ageing. Upon physiological ageing, MSCs exhibit skewed differentiation at the expense of osteoblasts, which leads to accumulation of adipocytes (upper panel). Treatment of aged MSCs with acetate, α-KG or 5-azacytidine (5-AzaC) enhances stemness and re-establishes a balanced differentiation potential, via altering the stem cell epigenome.

One of the first studies analysing global changes in the DNA methylation profile of purified HSCs from young and old mice used reduced representation bisulfite sequencing (RRBS) [74]. The authors found that the DNA methylation pattern is fairly stable upon ageing, with only a slight increase in residue-specific DNA methylation levels, particularly in sites associated with the stem cell haematopoietic capacity. This study also revealed that ageing in HSCs is linked to DNA hypermethylation at genes that are regulated by the polycomb repressive complex 2 (PRC2). Importantly, these findings were confirmed later by an independent study [65]. Recently, also human HSCs were profiled using RRBS [66]. Of the ~3 million identified CpGs sites, around ~2200 sites changed their DNA methylation status upon ageing. Interestingly, in the same study the authors investigated age-related changes in histone modifications and integrated these results to differentially methylated DNA regions at functional sites of the genome, such as enhancers. Data analysis revealed a strong change in promoter and enhancer DNA methylation of genes that are involved in developmental processes and cancer. Remarkably, these genes are also affected in a similar manner in acute myeloid leukaemia (AML) [66], indicating that the ageing DNA methylation pattern resembles a tumour-like state. These studies on HSCs propose that the DNA methylation profile changes with age. However, these changes are subtle and affect only a subset of sites, suggesting that overall DNA methylation is fairly stable. Likewise, single-cell DNA methylation analysis of MuSCs revealed that the DNA methylation profile remains relatively unchanged upon ageing with only modest increase of DNA methylation, particularly over SINE elements and regions marked by H3K36me3 [75]. However, it is important to highlight that the examples discussed above refer to studies that were carried out in quiescent stem cells. Results of genomic experiments might be different in other compartments, in which stem cells divide more frequently, such as the intestine, in which DNA methylation changes are sufficient to predict the donor’s age using epigenetic clocks [76].

In contrast to DNA methylation, histone modifications undergo profound alterations during ageing in various types of somatic stem cells. In human HSCs, ChIP-seq analysis revealed a strong reduction of H3K4me1, H3K27ac and H3K4me3 levels upon ageing, while H3K27me3 levels are only mildly affected [66]. In contrast, aged murine HSCs display a significant increase in H3K4me3 and H3K27me3 levels in comparison to the young HSCs. In addition to its altered levels, H3K4me3 mark in aged HSCs exhibits a broader distribution over regions associated with HSC identity and self-renewal [65]—a phenomenon that is thought to enhance transcriptional consistency and increase transcriptional output [77]. On the other hand, MuSCs are characterized by a global loss of H3K4me3 and a slight increase of H3K27me3 levels [71]. Importantly, in all of the described cases, the epigenetic changes were corroborated by transcriptional alterations. We recently performed ATAC-sequencing on MSCs isolated from young and old mice and observed a strong decrease in chromatin accessibility, which is in agreement to a global histone hypoacetylation in aged MSCs [69], contrary to the belief that generally, chromatin becomes more accessible with age.

Together, these data point towards a cell-type-specific change in the chromatin modification pattern with age, and they highlight the necessity for a deeper understanding of the contribution of histone modifications to the ageing process of stem cells. Integration of the histone modification profile with chromatin accessibility patterns, the DNA methylation distribution and the transcriptional output will hopefully help us generate a more complete view regarding the precise mechanisms via which the epigenome regulates ageing in somatic stem cells.

## Outlook: interfering with the epigenome to positively impact longevity

Although we have only recently started to study the changes that occur in the epigenome of ageing stem cells, it is clear that chromatin structure plays a fundamental role in regulating stem cell fate and function. Interestingly, since modulation of the chromatin architecture enables targeted alteration of the transcriptional output and ultimately of the stem cell identity, the epigenome represents an attractive target for development of anti-ageing strategies (Figure 2). Of note, such approaches to direct stem cell fate decisions towards specific lineages could be combined with the currently used approaches during autologous stem cell therapy. For instance, *ex vivo* manipulation of the chromatin landscape to enhance stem cell activity prior to HSC and MSC transplantation could potentially increase the efficiency of stem cell therapies. Proof-of-concept studies have been published in the last years using model organisms to explore the potentially beneficial effects of such approaches. For example, *ex vivo* treatment of aged MuSCs using a combination of biophysical (rigidity of culture matrix) and biochemical (p38a/b inhibitor) approaches resulted in MuSC rejuvenation. Indeed, after transplantation, treated MuSCs contributed to extensive myofiber repair and restored strength to injured muscles in aged mice [78]. While this study did not use any epigenetic intervention, it clearly illustrates the potential of an *ex vivo* therapy. On the other hand, inhibition of DNMTs by 5-azacytidine has been used in various studies to enhance stem cell differentiation capacity of MSCs purified from elderly human donors [79, 80]. Furthermore, two recent studies demonstrated that supplementation of MSCs with the epigenetic-related metabolites a-KG and acetate improves osteogenesis in aged MSCs. Mechanistically, both metabolites elicit changes on chromatin architecture; in particular, a-KG leads to decreased repressive marks on the promoters of osteogenic genes [81], whereas acetate restores histone acetylation levels and thus, chromatin accessibility on osteogenesis-involved genes [69]. In line to this, manipulating the intracellular metabolism by regulation of nutrient-sensing pathways via caloric restriction and/or pharmacological interventions, such as rapamycin and resveratrol treatment, influences stem cell fate decisions, via altering the epigenome. Therefore, *ex vivo* treatments to manipulate the chromatin landscape are emerging as a potential tool for efficient stem cell rejuvenation.

Although there has been significant progress in the use of epigenetic interventions in various cancer types [82], before we start following such approaches to delay age-related decline in stem cell function, we should address several key-issues, including (i) a more complete understanding of the profile of epigenetic modifications in ageing stem cells and the underlying molecular mechanisms and (ii) the development of safe and efficient protocols for stem cell purification and transplantation.

**Key Points**

- The epigenetic state of a stem cell is tightly linked with cellular fate.
- Ageing leads to changes on the epigenetic level and thus, alters stem cell potential and fate.
- Targeting the ageing epigenome might represent a potent strategy to re-establish stem cell potency in the elderly and to extend health-span.

## Notes

Andromachi Pouikli is a graduate student at the MPI for Biology of Ageing. She is interested in understanding the connection between metabolism and epigenetics during stem cell ageing.

Peter Tessarz is a group leader at the MPI for Biology of Ageing. His main interest lies in understanding how chromatin-mediated regulation of transcription is impacted by ageing.
